# Reproducibility of a combined artificial intelligence and optimal-surface graph-cut method to automate bronchial parameter extraction

**DOI:** 10.1007/s00330-023-09615-y

**Published:** 2023-04-18

**Authors:** Ivan Dudurych, Antonio Garcia-Uceda, Jens Petersen, Yihui Du, Rozemarijn Vliegenthart, Marleen de Bruijne

**Affiliations:** 1 Department of Radiology, University Medical Centre Groningen, University of Groningen, Groningen, Netherlands; 2 Department of Radiology and Nuclear Medicine, Erasmus MC, BIGR - Na 26-20, Doctor Molewaterplein 40, 3015 GD Rotterdam, Netherlands; 3 Department of Paediatric Pulmonology and Allergology, Erasmus MC-Sophia Children Hospital, Rotterdam, Netherlands; 4 Department of Computer Science, University of Copenhagen, Copenhagen, Denmark; 5 Department of Epidemiology, University Medical Centre Groningen, University of Groningen, Groningen, Netherlands; 6 Data Science in Health (DASH), University Medical Centre Groningen, University of Groningen, Groningen, Netherlands

**Keywords:** Computed tomography, X-ray, Thorax, Bronchi, Artificial intelligence

## Abstract

**Objectives:**

Computed tomography (CT)–based bronchial parameters correlate with disease status. Segmentation and measurement of the bronchial lumen and walls usually require significant manpower. We evaluate the reproducibility of a deep learning and optimal-surface graph-cut method to automatically segment the airway lumen and wall, and calculate bronchial parameters.

**Methods:**

A deep-learning airway segmentation model was newly trained on 24 Imaging in Lifelines (ImaLife) low-dose chest CT scans. This model was combined with an optimal-surface graph-cut for airway wall segmentation. These tools were used to calculate bronchial parameters in CT scans of 188 ImaLife participants with two scans an average of 3 months apart. Bronchial parameters were compared for reproducibility assessment, assuming no change between scans.

**Results:**

Of 376 CT scans, 374 (99%) were successfully measured. Segmented airway trees contained a mean of 10 generations and 250 branches. The coefficient of determination (*R*^2^) for the luminal area (LA) ranged from 0.93 at the trachea to 0.68 at the 6^th^ generation, decreasing to 0.51 at the 8^th^ generation. Corresponding values for Wall Area Percentage (WAP) were 0.86, 0.67, and 0.42, respectively. Bland–Altman analysis of LA and WAP per generation demonstrated mean differences close to 0; limits of agreement (LoA) were narrow for WAP and Pi10 (± 3.7% of mean) and wider for LA (± 16.4–22.8% for 2–6^th^ generations). From the 7^th^ generation onwards, there was a sharp decrease in reproducibility and a widening LoA.

**Conclusion:**

The outlined approach for automatic bronchial parameter measurement on low-dose chest CT scans is a reliable way to assess the airway tree down to the 6^th^ generation.

**Statement on clinical relevance:**

This reliable and fully automatic pipeline for bronchial parameter measurement on low-dose CT scans has potential applications in screening for early disease and clinical tasks such as virtual bronchoscopy or surgical planning, while also enabling the exploration of bronchial parameters in large datasets.

**Key Points:**

• *Deep learning combined with optimal-surface graph-cut provides accurate airway lumen and wall segmentations on low-dose CT scans*.

• *Analysis of repeat scans showed that the automated tools had moderate-to-good reproducibility of bronchial measurements down to the 6*^*th*^ *generation airway*.

• *Automated measurement of bronchial parameters enables the assessment of large datasets with less man-hours*.

**Supplementary Information:**

The online version contains supplementary material available at 10.1007/s00330-023-09615-y.

## Introduction

Bronchial parameters are increasingly being investigated for use in the characterisation of pulmonary diseases such as chronic obstructive pulmonary disease (COPD) [1]. A potential benefit of developing robust bronchial parameters is the early detection of pulmonary disease. For example, screening for lung cancer with computed tomography (CT) may offer the opportunity for the evaluation of “off-target” organ systems such as the heart, bronchi, and vasculature [2]. While bronchial parameters could be used for the evaluation of pulmonary disease, their use is limited by the man-hours necessary for (manual) measurements. This step is further complicated by the low dose of screening CT scans, which can result in a worse image quality with more noise. Due to this, the development of reliable automated methods for CT bronchial parameter measurement is a necessary step.

To calculate bronchial parameters, most methods require segmenting and measuring the airway lumen and wall from chest CT scans. Segmentation of the airway lumen is challenging, due to the complex structure of the airway tree and the small size of most branches. Recently, deep learning methods for automatic segmentation of the airway lumen have achieved success [3–7]. Segmentation of the airway walls in the smaller branches is even more demanding, due to its small thickness and low contrast between the wall, lumen, and surrounding parenchyma. The thickness of the wall may fall below the scanner resolution, therefore lacking the stark contrast available in the larger airways. Airway wall segmentation has received less attention; currently, there are no automatic methods to obtain this directly from the CT scan without an initial seed placement or lumen segmentation [8]. Instead, the airway wall can be obtained as an additional refinement step using, for example, full-width at half-maximum [9], phase congruency [10], or optimal-surface graph-cut methods [11].

To evaluate early biomarkers of respiratory disease on low-dose chest CT scans, we built an automated pipeline for segmenting and quantifying the airway lumen and wall. We did this by combining two validated, open-source methods, for obtaining the airway lumen and wall segmentations, respectively. While previous studies have evaluated AI on lumen segmentations, we could not identify studies that have assessed their reproducibility when also measuring the airway wall in a fully automated way. Furthermore, this is the first combination of these 3D-Unet and 3D optimal-surface graph-cut methods for fully automated bronchial parameter evaluation. We aim to quantify the repeatability of this pipeline on low-dose chest CT. Subsequently, we computed the bronchial parameter measurements. We tuned this pipeline for the low-dose chest CT scan protocol and investigated its reproducibility using short-term repeated scanning.

## Methods

### CT scans

Scans for this study were obtained from the Imaging in Lifelines (ImaLife) study, which was approved by the local medical ethics committee, and is registered with the Dutch Central Committee on Research Involving Human Subjects (https://www.toetsingonline.nl; Identifier: NL58592.042.16).

All scans were obtained using third-generation dual-source CT (Somatom Force, Siemens Healthineers). Imaging was performed with the participants in a supine position and coached to hold their breath at maximum inspiration. The ImaLife scanning protocol for lung imaging was as follows: 120 kVp, 20 mAs, pitch 3.0 (2.5 in large habitus), 1/0.7 mm slice thickness/increment, and dose length product (DLP) of < 100 mGycm. Images were reconstructed with a quantitative-sharp reconstruction kernel (Qr59) [12].

### Lumen segmentation

We used a deep learning airway segmentation method (Bronchinet) [3], based on a 3D U-Net model, to automatically obtain airway lumen segmentation from the CT scans. For training, we used a dataset of 24 ImaLife scans to train Bronchinet from scratch, with ground truth airway segmentations generated with a previously reported method [13]. From the full dataset, we used 22 scans for training (i.e., optimising the model weights) and the remaining 2 scans for validation (i.e., early stopping and model convergence). The Bronchinet method was validated in a previous paper with a training set of similar size showing good performance [3]. We assessed the model performance using sixfold cross-validation.

### Wall segmentation

An optimal-surface graph-cut method (Opfront) [11, 14] was used to refine the Bronchinet airway lumen segmentations and obtain the wall segmentation. Opfront performance was tuned on several parameters, which depend on the scan resolution and protocol. We optimised the Opfront parameters using the COPDGene phantom, scanned using the ImaLife protocol [15]. The optimised parameters were inner and outer derivatives, smoothness penalties/constraints, and surface separation penalty. These parameters were focused on as they most strongly influence the resulting lumen and wall segmentation. For all other parameters, we used the values suggested in Petersen (2015) [11]. The lumen and total diameters of the Opfront segmentation for the phantom tubes were measured and compared to the known dimensions. To automatically search for the optimal parameter values, we used the Tree-structured Parzen Estimator algorithm [16], which modified parameters if the measurement error between phantom measurements and known dimensions was large [11]. Once phantom measurements were close to the known dimensions, Opfront was considered optimised.

### Measurement of branches

From the airway lumen segmentations obtained by Bronchinet, discarding disconnected components. individual branches were extracted using a front-propagation method as described in the EXACT’09 challenge [17, 18]. Branch generations were determined based on Weibel’s airway model, which defines a new generation at each branching point [19]. Measurements of the airway lumen and wall radii were calculated for all branches, measured at regular intervals of 0.5 mm along the branch centreline, and averaged. Terminal branches of less than 2 mm in length were automatically discarded.

### Automated pipeline

We combined the Bronchinet and Opfront methods in an automated pipeline to obtain the wall segmentation and bronchial parameter measurements directly from input CT scans. For this, we built a docker image [20] to link both tools and manage software dependencies. This allows deploying the pipeline in any computing system featuring at least 16 GB RAM and a CUDA-compatible graphics card with at least 8 GB memory.

### Reproducibility study

A total of 188 ImaLife participants with two scans an average of 3 months apart were included. None of these participants were included in the Bronchinet model training. For more information on the ImaLife study, please see prior publications [12, 21]. Participants were invited for a short-term repeat scan for scientific purposes in case of an intermediate nodule (100–300 mm^3^) on the first scan. All scans were automatically processed by the proposed pipeline. Inspiration levels were quantified based on the total lung volume (TLV), derived from automated lung segmentation [22]. Participants with a difference in inspiration defined by a TLV difference between first and second scans greater than 15% were excluded from the analysis [23]. Bronchial parameters were automatically calculated from airway branch lumen and wall radii, namely luminal area (LA), wall area percentage (WAP), and the square root of the wall area (SRWA) at a hypothetical airway with an internal perimeter of 10 mm (Pi10). Pi10 was calculated by linear regression of SRWA compared to the internal perimeter of the airway branch, excluding the trachea, and including branches up to and including the 6^th^ generation (Figure S1) [24].

### Statistical analysis

To measure the reproducibility of the pipeline, the coefficient of determination (*R*^2^) was calculated for bronchial parameters per airway generation by first and second CT scan comparison. An *R*^2^ of > 0.7 was considered good, 0.7–0.5 moderate, and < 0.5 poor [25]. Bland–Altman analysis was performed to calculate the limits of agreement (LoA) for each bronchial parameter per generation. The python package statsmodels (v 0.13.5) was used for statistical analysis [26].

## Results

### Bronchinet and Opfront performance

On the cross-validation assessment of Bronchinet with the 24 ImaLife scans, the median Dice overlap coefficient for the obtained airway lumen segmentations was 0.92 (inter-quartile range (IQR), 0.83–0.93), the median centreline completeness was 85.2% (IQR 78.8–89.4%), and the median centreline leakage (indicating predicted false-positive centrelines) was 7.1% (IQR, 3.5–10.8%) As the volume of the trachea and main bronchi dominate these values, they were excluded to focus on downstream segmentation performance.

**Fig. 1:**
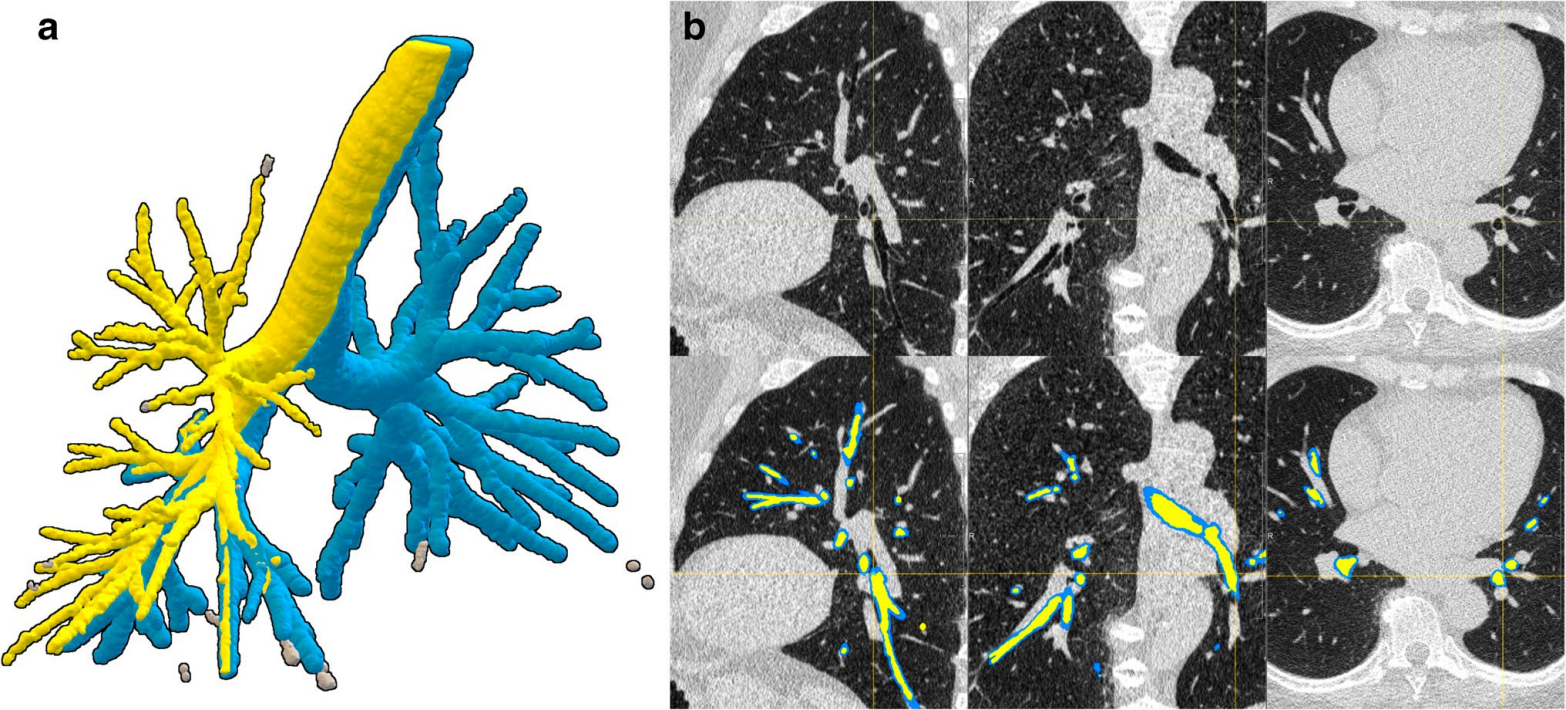
**a** 3D rendering of the airway lumen (yellow) and wall (blue) of an ImaLife participant CT scan. Disconnected components (grey) were discarded prior to bronchial parameter measurement. Maximum generation: 8. **b** 2D overlay of the airway lumen (yellow) and wall (blue) segmentations on sagittal, coronal, and axial planes respectively

The optimised Opfront segmentation of the COPDGene phantom resulted in sub-voxel accuracy. The lumen diameter was estimated within a mean unsigned error of 3.1% (0.13 ± 0.07 mm), and the total diameter with an average unsigned error of 5.8% (0.35 ± 0.20 mm) (Table 1). The airway segmentations were fully 3D and the extracted airways reached the 10^th^ generation on average (Fig. 1). The total execution time was 28 ± 4 min per scan.

**Table 1:** Opfront measurement error for phantom tube lumen and wall

	Measurement error (mm)	
Tube	Lumen	Wall
1	0.09	− 0.15
2	0.09	0.03
3	0.09	− 0.22
4	− 0.00	0.21
5	0.05	− 0.01
6	0.04	0.24
7	− 0.05	0.33
8	0.14	− 0.22

### Reproducibility study

Out of 376 scans, 374 (99%) were successfully segmented and measured. Twenty of 188 participants were excluded due to a difference in TLV of > 15% between the first and second scans. The final group comprised 98 male and 70 female participants with a repeat scanning within 3 months (98 ± 14 days). The mean age was 59.6 ± 9.4 and the body mass index (BMI) was 26.5 ± 3.8. Of the 168 included participants, 40 were never-smokers, 76 were smokers, 39 had a COPD diagnosis and 13 participants had missing COPD disease status (Table 2). The mean pack-year history for smokers was 14.7 ± 8.1 years. Mean CT measurements were 5.52 ± 1.28 L for TLV, 250 ± 54 for total airway count (TAC), 42.0 ± 2.3% for 3^rd^ generation LA, 56.4 ± 3.4% for 3^rd^ generation WAP, and 3.92 ± 0.12 for Pi10.

**Table 2:** Characteristics of participants

Variable	Number (%) or Mean ± SD
Participants	168 (100%)
Male/female	98 (58%) /70 (42%)
Never-smoking	40 (24%)
Smoking	76 (45%)
COPD	39 (23%)
No status	13 (8%)
Age (years)	59.6 ± 9.4
BMI (kg/m^2^)	26.46 ± 3.78
Pack-years*	14.7 ± 8.1
TLV (L)	5.52 ± 1.28
Pi10 (mm)	3.92 ± 0.12
WAP (%)	56.4 ± 3.42
LA (mm^2^)	42.0 ± 2.31
TAC (*n*)	250 ± 54

Data are displayed as mean and standard deviation or number (percentage). Mean CT measurements were calculated from the first scan. *BMI* body mass index, *N* number, *SD* standard deviation, *TLV* total lung volume, *WAP* wall area percentage, *LA* luminal area, *TAC* total airway count. * Pack-years do not include never-smokers

**Fig. 2:**
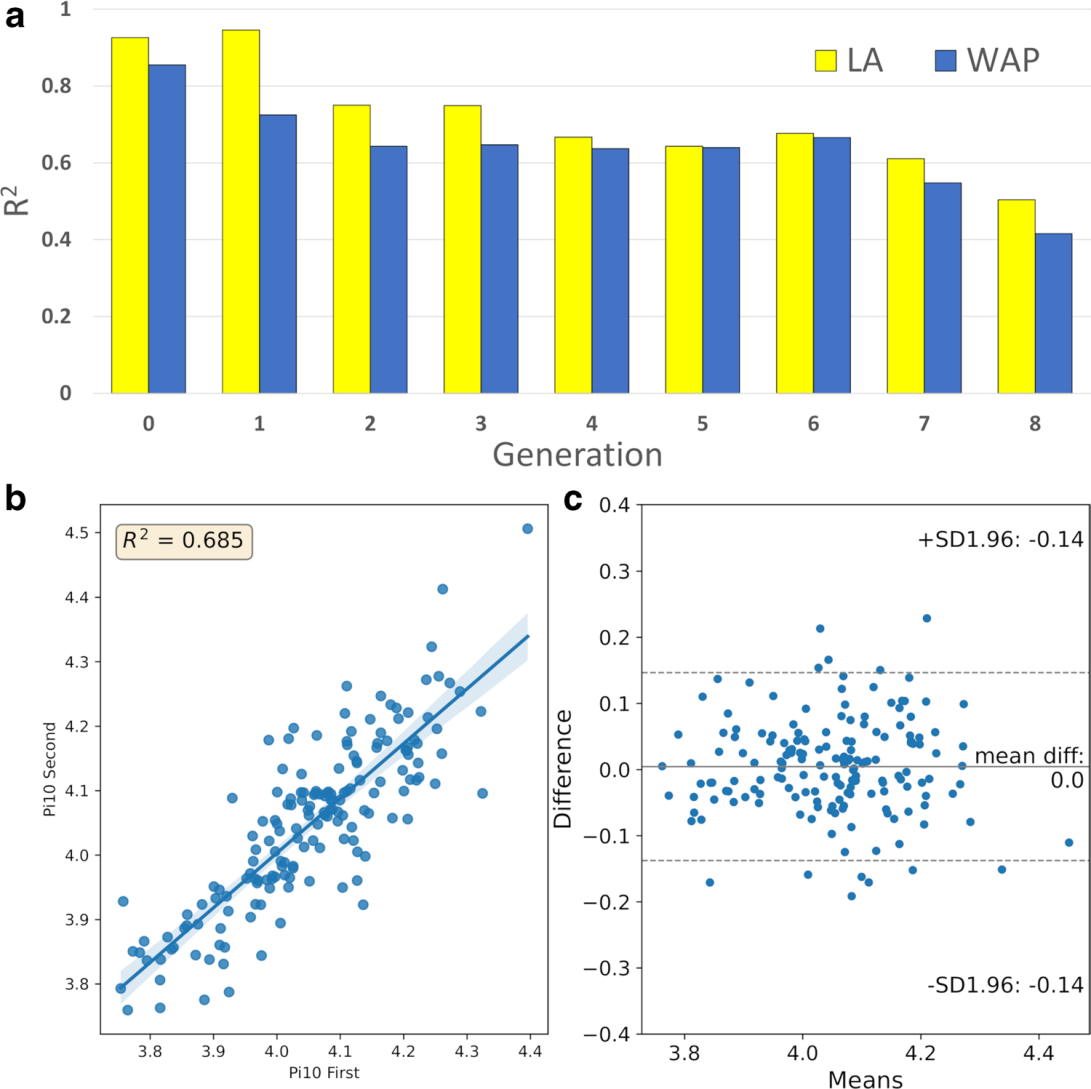
**a** Reproducibility analysis. Comparison of bronchial parameter measurements between first and second scans per generation by the coefficient of determination. **b** Scatter plot and regression line of Pi10 measurement on first and second scans. **c** Limits of agreement for Pi10 between first and second scans. *R*^2^ coefficient of determination, *SD* standard deviation

The coefficient of determination (*R*^2^) of LA ranged from 0.93 at the trachea to 0.68 at the 6^th^ generation, decreasing to 0.51 at the 8^th^ generation. Corresponding values for WAP were 0.86, 0.67, and 0.42, respectively (Fig. 2A).

For Pi10, *R*^2^ was 0.69 (Fig. 2B) and LoA was ± 0.14 mm (± 3.7% of mean) with a mean difference (MD) of 0.00 mm (Fig. 2C). For LA, MD ± LoA ranged from − 0.1 ± 37 mm^2^ at the trachea to − 0.1 ± 3.7 mm^2^ at the 6^th^ generation, and down to − 0.15 ± 6.6 mm^2^ at the 8^th^ generation (Table 3). For WAP, MD ± LoA ranged from 0.05 ± 1.4% at the trachea to 0.14 ± 4% at the 6^th^ generation and down to 0.25 ± 5.4% at the 8^th^ generation. LoA expressed as a percentage of the mean (LoA%) was between ± 5.9 and 9.3% for WAP. LoA% for LA was ± 7.4–6.8% at the 0–1^st^ generations, widening to ± 16.4–22.8% for the 2^nd^ to 6^th^ generations, and further increasing to ± 28.9–36.3% at the 7^th^ to 8^th^ generations (Table 3).

**Table 3:** Mean difference (MD) and limits of agreement (LoA) and LoA as a percentage of overall range (LoA%) between the first and second scan for the luminal area and wall area percentage per airway generation

	Luminal area (mm^2^)			Wall area percentage (%)		
Gen	MD	LoA	LoA%	MD	LoA	LoA%
0	− 1.10	± 37	± 7.4	0.05	± 1.4	± 5.9
1	− 0.19	± 16	± 6.8	0.02	± 2.3	± 6.1
2	0.37	± 19	± 16.4	− 0.14	± 3.8	± 6.1
3	0.19	± 9.2	± 12.6	− 0.01	± 4.2	± 9.1
4	0.25	± 6.3	± 20.6	− 0.13	± 4.5	± 8.5
5	− 0.09	± 4.4	± 22.5	0.12	± 4.2	± 7.5
6	− 0.10	± 3.7	± 22.8	0.14	± 4.0	± 6.8
7	− 0.04	± 3.5	± 28.9	0.12	± 4.5	± 7.4
8	− 0.15	± 6.6	± 36.3	0.25	± 5.4	± 9.3

*Gen* generation

## Discussion

In this study, we built an automated pipeline for low-dose chest CT scans to obtain segmentations of the airway lumen and wall by combining two open-source methods. The resulting segmentations yielded automated quantitative bronchial parameters. Repeated scans showed moderate to good reproducibility (*R*^2^ > 0.6) of bronchial parameters down to the 6^th^ generation. The Bland–Altman analysis showed no systematic bias and narrow limits of agreement for Pi10 and WAP, but wider for LA, demonstrating a lower variability in summary parameters like Pi10 and WAP compared to the direct measurement of LA.

The use of low-dose CT scans for lung cancer screening provides the opportunity to screen for other early diseases such as COPD, bronchiectasis, and cardiac disease, which may influence lung cancer risk and/or prognosis. Automated bronchial parameter measurement can enable the screening of large cohorts in a reasonable timeframe with good reliability. Furthermore, fully 3D segmentation can be readily useful in clinical tasks such as virtual bronchoscopy or surgical planning. However, for bronchial parameters, it is hard to determine whether the airways are normal or abnormal. The number of never-smokers in bronchial parameter research is typically very small [27]. Combined with heterogenous bronchial parameter methodology, it is unclear what quantitatively defines “normal” airways on low-dose CT and by which bronchial parameter. This study demonstrated a wider variability in measurements for LA than Pi10 or WAP. While this could in part concern variability or error due to methodology, additional factors like seasonal changes, smoking, or illness before a scan could result in true differences. Pi10 averages many branches, while WAP includes wall thickness in its calculation and so could be more resistant than LA to localised variations in measurements. Our pipeline provides similar reproducibility of LA and WAP as previous methods on similar datasets [11], but it also gives better reproducibility of Pi10 [28]. Additionally, it offers fully automatic bronchial parameter measurement using low-dose noisy scans.

Various methods can be used as an initial step for lumen segmentation. We used Bronchinet due to its state-of-the-art performance [3], speed, and open-source availability which enabled retraining on the low-dose scans in this study. Fully automated bronchial parameter calculation has been previously proposed using tools trained on manually traced borders alongside older algorithms such as FWHM, intensity-based, and phase congruency [29, 30]. However, previous research shows that manual and FWHM measurement overestimates the airway wall [31], which is also evident when used to measure the COPDGene phantom (Table S2). Compared to these approaches the advantage of our method is that Opfront was optimised on a phantom with precise physical measurements, eliminating the bias in wall measurements that comes with the previously mentioned approaches. The pipeline output is a ready-to-use 3D model of the airways, which has potential applications in tasks such as virtual bronchoscopy, airflow simulation, and 3D printing. Deploying the pipeline in a docker image provides the method as ready-to-use and implementable in clinical practice. For lumen segmentation, good results could be readily achieved by using the publicly available trained model bundled with Bronchinet [3], which uses airway segmentations for training from the Danish Lung Cancer Screening Trial [32] in combination with an Erasmus-MC Sophia (cystic fibrosis) dataset [33]. The ImaLife scan protocol has a lower radiation dose with a total DLP of < 100 mGycm, and more noise in the scans; retraining the tools resulted in better performance [13]. For maximum performance on different datasets, optimising the pipeline for the target CT protocol may be necessary. This was achieved by re-training the Bronchinet with efficiently generated ground truths, and tuning Opfront using a physical phantom.

A limitation of this study is the lack of severe airway disease in the cohort as the ImaLife study comprises a general population. Evaluation of severe cases is important prior to adoption in a clinical setting, where scan protocol may also change. For the analysis, we assumed that there are no short-term differences in bronchial parameters between the scans. However, factors such as illness or smoking before the scan could have an impact on the bronchial parameter results. This would tend to increase variability between scans, which could mean that the actual scan-rescan repeatability may be better than we currently report. The methods used do not perform anatomical airway labelling, and so we could not compare the repeat measurements of specific airway branches directly. Instead, we focused on average values per generation for participants. Lastly, Bronchinet does not guarantee a fully connected airway segmentation, some peripheral branches may be discarded during measurement. For cases with an occluded lumen, this could result in the exclusion of segmented airways beyond the blockage.

In conclusion, we demonstrate a comprehensive and fully automatic pipeline for bronchial parameter measurement on low-dose CT using open-source tools. Based on the results of short-term repeat CT scanning, the pipeline provides reliable bronchial parameters down to the 6^th^ generation. Overall, these methods enable the exploration of bronchial parameters in large low-dose CT datasets after an initial investment in the training and optimisation of deep learning and optimal-surface graph-cut methods.

### Supplementary Information

Below is the link to the electronic supplementary material.

Supplementary file1 (PDF 180 KB)

## Abbreviations

AI: Artificial intelligence
COPD: Chronic obstructive pulmonary disease
CT: Computed tomography
LA: Luminal area
LoA: Limits of agreement
LoA%: Limits of agreement as percentage of overall measurement range
Pi: Internal perimeter
Pi10: Square root of the wall area of a hypothetical airway with internal perimeter of 10 mm
R^2^: Coefficient of determination
SRWA: Square root of the wall area
TAC: Total airway count
TLV: Total lung volume
WAP: Wall area percentage

## Declarations

### Guarantor

The scientific guarantor of this publication is Prof. Marleen de Bruijne.

### Statistics and biometry

No complex statistical methods were necessary for this paper.

### Methodology

• Retrospective

• Experimental

• Performed at one institution
