# Supplementary material for: Reproducibility of a combined artificial intelligence and optimal-surface graph-cut method to automate bronchial parameter extraction
Source: Eur Radiol. 2023 Apr 18;33(10):6718–25. doi: 10.1007/s00330-023-09615-y (PMC10511366; doi:10.1007/s00330-023-09615-y)
Supplement: Supplementary file 1 — Supplementary file1 (PDF 180 KB) [file 330_2023_9615_MOESM1_ESM.pdf]

ELECTRONIC SUPPLEMENTARY MATERIAL

Reproducibility of a Combined Artificial Intelligence and Optimal-Surface Graph-Cut Method to Automate Bronchial Parameter Extraction

Supplementary Table S1

Tube	Real WT (mm)	FWHM WT (mm)	Error (mm)	Error (%)
1	0.4	0.94±0.37	+0.54	+135
2	1.2	1.32±0.19	+0.12	+10
3	1.2	1.45±0.17	+0.25	+21
4	0.4	1.02±0.16	+0.62	+155
5	0.9	1.22±0.03	+0.32	+36
6	0.6	1.15±0.0	+0.55	+92
7	0.6	1.17±0.03	+0.57	+95
8	1.5	1.62±0.06	+0.12	+8

Wall thickness (WT) measurement on the COPDGene phantom using the Full-Width Half-Maximum (FWHM) method, in comparison to the real dimensions of the phantom airways. FWHM ± Standard Deviation (SD).

Supplementary Figure S1

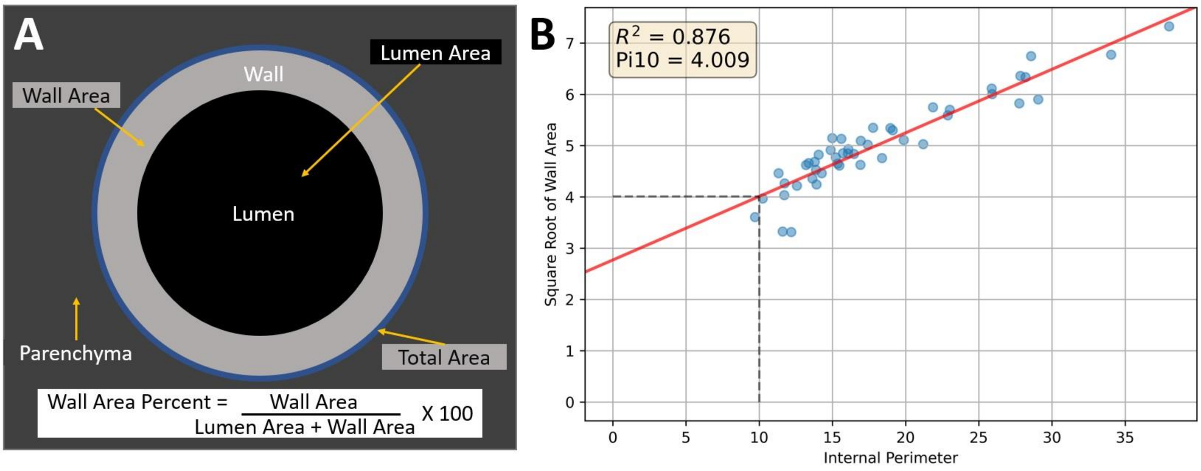

(A) Diagram illustrating the cross-section of an airway, from which bronchial parameters can be calculated. Luminal Area (LA) is calculated using the average lumen radius. The Total Area (TA) uses the average outer wall radius. Wall Area (WA) is calculated by subtracting the LA from TA. Wall Area Percent is calculated by dividing WA by TA and multiplying by 100.

(B) Example graph of Square Root of the Wall Area vs internal perimeter in mm for the purpose of calculating Pi10. Each data point is a single branch from the airway tree. Only branches up to 6th generation were included for Pi10 calculation.
